# Saliva-permeable and antimicrobial potentiometric pH sensor for oral health monitoring

**DOI:** 10.1016/j.isci.2026.114703

**Published:** 2026-01-15

**Authors:** Luyue Zhang, Mengya Wang, Liting Xiao, Xinchang Li, Ziqi Sha, Feng Yang

**Affiliations:** 1 School of Stomatology, Xuzhou Medical University, Xuzhou 221004, China; 2 Department of Stomatology, The Affiliated Hospital of Xuzhou Medical University, Xuzhou 221002, China; 3 School of Mechanical Engineering and Automation, Northeastern University, Shenyang 110004, China; 4 WEGO JERICOM Biomaterials Co., Ltd, Weihai 264210, China; 5 School of Life Science, Xuzhou Medical University, Xuzhou 221004, China

**Keywords:** Bioengineering, materials science engineering

## Abstract

Oral health hinges on tightly regulated acid-base chemistry that fluctuates within minutes after eating or hygiene, yet existing assessments are episodic and miss these transient events. We demonstrate a liquid-permeable potentiometric pH sensor designed for non-invasive intraoral monitoring that integrates a saliva-through fibrous layer for fast convective-capillary transport, a percolated silver-nanowire network for low-impedance readout and antibacterial activity, and a pH-responsive sensing layer paired with an on-board Ag/AgCl reference. The integration sensor delivers near-Nernstian sensitivity, rapid and reversible responses, and low drift during extended operation under simulated oral conditions. Microbiological assays show the inhibition of Streptococcus mutans and Candida albicans, supporting resistance to biofilm fouling. Moreover, in human-use demonstrations, the sensor captures real-time salivary pH excursions during sugar intake, mixed meals, and tooth brushing. This platform establishes a practical route toward patient-centric oral electronics that shift diagnosis and prevention from the clinic into everyday life.

## Highlights

• Saliva-permeable antibacterial pH sensor enables real-time intraoral monitoring
• Near-Nernstian sensitivity with fast, reversible, and drift-free pH response
• Captures rapid oral pH dynamics for caries-risk and hygiene evaluation

## Graphical abstract

**Figure:**
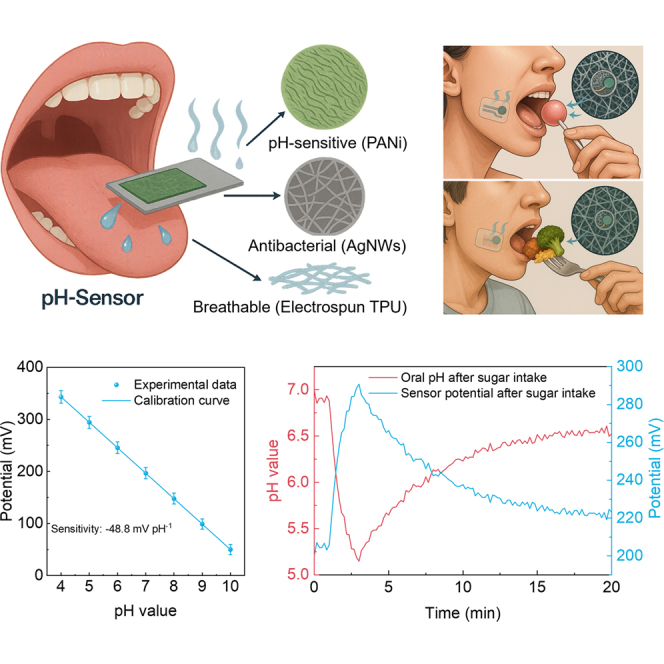

## Introduction

Oral health is fundamental to overall well-being, influencing nutrition, speech, social interaction, and systemic conditions such as cardiovascular and metabolic diseases.^1^^,^^2^^,^^3^ The oral cavity hosts a dynamic ecosystem in which saliva, biofilms, and a complex microbiota interact under tightly regulated biochemical conditions. Disruptions to this balance, often triggered by dietary sugars, microbial colonization, or changes in salivary composition, can lower local pH, driving enamel demineralization, altering microbial ecology, and initiating pathologies such as dental caries, gingivitis, and periodontitis.^4^^,^^5^^,^^6^ Globally, oral diseases remain among the most prevalent health conditions, imposing significant social and economic burdens. Acid-base balance, reflected in salivary and plaque pH, is a key early indicator of oral health.^7^^,^^8^ Monitoring the dynamics of salivary pH, not only absolute values, is clinically meaningful. Rapid acidification after sugar intake and delayed pH recovery are strongly linked to enamel demineralization rates, microbial dysbiosis, and caries risk. Continuous, real-time pH tracking, therefore, provides clinically actionable information that cannot be captured by single-time-point measurements.^9^ Rapid pH fluctuations occur within minutes after carbohydrate intake, preceding clinical signs by weeks or months. Continuous monitoring of these variations could enable early detection, personalized dietary guidance, and timely intervention.^10^^,^^11^^,^^12^ However, current pH assessment methods, visual inspection, spot salivary tests, and laboratory analysis are episodic, operator-dependent, and unable to capture transient biochemical events.^13^^,^^14^ Wearable chemical sensors have emerged as a transformative technology for non-invasive, real-time health surveillance, enabled by advances in flexible electronics, biocompatible materials, and low-power, miniaturized signal processing.^15^^,^^16^^,^^17^^,^^18^^,^^19^^,^^20^ Such devices can be seamlessly integrated into daily life, continuously tracking physiological and biochemical parameters to enable early diagnosis, personalized treatment, and dynamic health management.

Platforms designed for skin-mounted monitoring, targeting biofluids such as sweat, tears, or interstitial fluid, have achieved remarkable sensitivity, specificity, and temporal resolution through optimized material-interface engineering, microfluidic control, and integrated wireless data transmission.^21^^,^^22^^,^^23^ However, extending these capabilities to the oral cavity introduces a unique set of engineering and biological challenges that exceed those encountered in epidermal or ocular applications. The intraoral environment is inherently harsh: surfaces are constantly wetted by saliva, subjected to rapid temperature fluctuations, and mechanically stressed by mastication, speech, and facial movement.^24^^,^^25^ Furthermore, the oral cavity hosts a dense and diverse microbial community that readily colonizes surfaces, forming biofilms capable of degrading sensor performance and compromising hygiene.^26^ An effective intraoral sensor must therefore integrate high biochemical responsiveness with long-term mechanical robustness, biocompatibility, and active resistance to biofouling.^27^^,^^28^ Beyond mechanical durability, such devices must contend with continuous exposure to saliva containing proteins, enzymes, electrolytes, and microbial metabolites, as well as intermittent contact with food debris and beverage residues.^29^^,^^30^ These factors drive rapid changes in local pH, ionic strength, and chemical composition, which can both inform diagnostic measurements and interfere with sensor accuracy. To address these constraints, an oral sensor must achieve selective permeability, permitting rapid analyte transport to the sensing interface, while simultaneously providing robust physical and chemical protection for its functional components, ensuring stable and reproducible operation over extended use.

Here we report a liquid-permeable, antimicrobial potentiometric pH sensor for continuous, non-invasive oral monitoring. The architecture follows three principles: (i) a saliva-through fibrous layer that enables rapid convective-capillary transport to the sensing interface; (ii) a percolated Ag nanowire (AgNW) network that affords low-impedance readout together with intrinsic antibacterial activity; and (iii) a pH-responsive sensing layer paired with an on-board Ag/AgCl reference to deliver near-Nernstian sensitivity (≈48.8 mV·pH^−1^) and stable operation (≥60 min). By combining permeability, antimicrobial protection, and electrochemical transduction in a single stack, the device exhibits fast, reversible responses across pH 4–10 with minimal drift during extended tests in simulated oral conditions. Standard microbiological assays show the robust inhibition of oral pathogens, including Streptococcus mutans and Candida albicans, indicating resistance to biofilm fouling. The same materials and system principles readily capture real-time salivary pH dynamics during sugar intake, meals, and tooth-brushing. These results establish a practical foundation for next-generation, patient-centric oral electronics that move diagnosis and prevention from the clinic into daily life.

## Results and discussion

### Design principles of a saliva-permeable pH sensor

**Figure 1:**
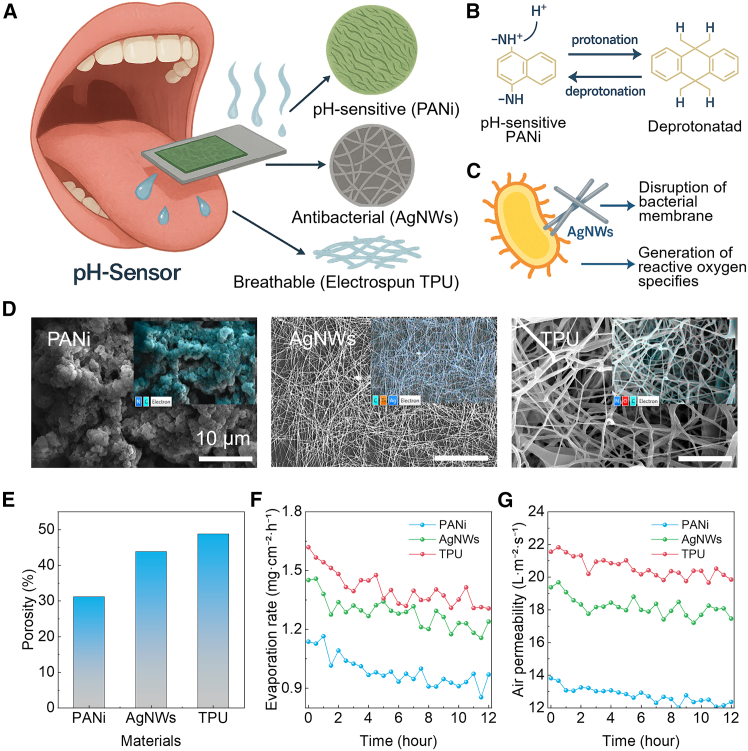
Saliva-permeable pH sensor: architecture, mechanisms, microstructure, and transport (A) Concept illustration of the intra-oral strip integrating a pH-sensitive PANi working electrode, an antibacterial and conductive AgNW, and a breathable electrospun TPU scaffold that permits saliva/vapor to pass through to the sensing interface. (B) Protonation/deprotonation of PANi (emeraldine base ⇄ salt) underlying the potentiometric response vs. Ag/AgCl. (C) Antimicrobial action of AgNWs by bacterial-membrane disruption and reactive-oxygen-species generation. (D) Representative SEM images of the wrinkled PANi surface, the percolated AgNW mesh, and the highly preamble electrospun TPU. (E) Open-porosity comparison for three scaffolds. (F) Water-vapor transmission over 12 h (30-min sampling) increases with porosity and shows only mild approach-to-steady-state decay. (G) Air permeability measured under a constant pressure differential likewise increases with porosity and remains stable over time.

We designed a saliva-permeable pH-sensor that integrates three functional layers, an electrospun TPU fiber as substrate for breathability, a screen-printed AgNW network for antibacterial protection and conductive connection, and a PANi sensing layer for proton-coupled transduction, into a thin, flexible strip suitable for intra-oral deployment (Figure 1A). Mechanistically, PANi responds to pH via reversible protonation/deprotonation of amine sites that modulate the material’s electronic structure and potential (Figure 1B), while AgNWs in direct contact with the oral milieu provide passive antimicrobial defense through membrane disruption and reactive-oxygen-species-mediated stress (Figure 1C). Thus, this material stack aims to sustain continuous saliva access to the sensing interface without compromising hygiene or comfort. Morphology and open-porosity analyses confirm that the fibrous substrate presents an interconnected void network that is only weakly occluded by the conductive and sensing layers. SEM micrographs (Figure 1D) and corresponding EDS mappings reveal a multi-scale architecture: (i) a rough, high-surface-area PANi topography that facilitates wetting and proton exchange; (ii) a percolated AgNW mesh forming long, tortuous channels between wires; and (iii) a highly porous TPU scaffold that underpins through-thickness mass transport. Quantification of open area by the Otsu segmentation of representative images yields three porosity states, 0.31, 0.44, and 0.49 for PANi, AgNWs, and TPU fiber (Figure 1E), spanning the range typically targeted for breathable, moisture-managing membranes while avoiding mechanical embrittlement. These values serve as the basis for correlating structure with transport. Next, we evaluated water evaporation for moisture management and saliva vapor handling. The evaporation rate (at 20 °C) scales monotonically with porosity across a 12 h series sampled every 30 min (Figure 1F). The average rate over the full test window increased from 0.99 mg cm^−2^ h^−1^ (PANi) to 1.28 mg cm^−2^ h^−1^ (AgNWs) and 1.41 mg cm^−2^ h^−1^ (TPU). The absolute values situate the sensor in a “breathable yet protective” regime appropriate for intra-oral wear, promoting rapid equilibration of the local microclimate and preventing liquid accumulation at the interface that could blur potentiometric readouts. Complementary air permeability measurements under a constant pressure differential (ΔP = 125 Pa, ASTM D737 style) reinforce the porosity-transport coupling (Figure 1G). The 12 h mean area-normalized flow increased from 12.78 L m^−2^ s^−1^ (PANi) to 18.20 L m^−2^ s^−1^ (AgNWs) and 20.59 L m^−2^ s^−1^ (TPU). Importantly, the increase in air permeability with porosity is sub-linear, consistent with a tortuosity-limited flow where additional void fraction provides diminishing returns once a percolated channel network is established. Together, water evaporation and air-permeability results demonstrate that the electrospun fiber governs mass transfer, while the AgNW/PANi stack preserves a large fraction of the substrate’s open area. The combined materials choice (TPU/AgNWs/PANi), porosity-guided transport tuning, and antibacterial current collection provide a coherent route to stable, breathable, and hygienic saliva pH sensing, aligning device architecture with the demands of continuous, real-world operation.

### Electrochemical performance of the potentiometric pH sensor

**Figure 2:**
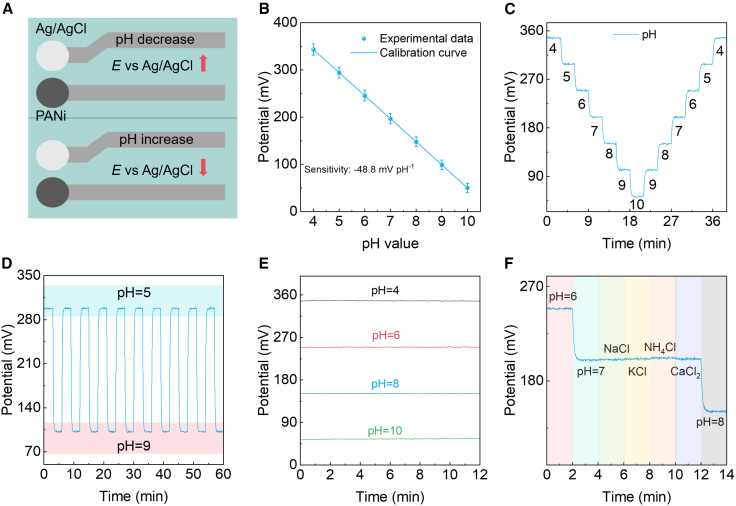
Saliva-permeable potentiometric pH sensor and electrochemical performance (A) Top-view device layout shows a PANi working electrode and an on-board Ag/AgCl reference. Arrows indicate the sign of the potential change E vs. Ag/AgCl upon pH decrease (up) and increase (down). (B) Calibration of E versus pH (4–10) shows a near-Nernstian slope of 49 mV pH^−1^ (mean ± s.d.; *n* = 6 sensors). Error bars denote s.d. (C) Continuous staircase response for a 60-min sequence (3-min holds; 4→10→4). (D) Cycling stability between pH 5 and pH 9 (6-min period; 60 min total). (E) Long-term stability at fixed pH (4, 6, 8, 10) over 12 h with 5-min sampling. (F) Selectivity at constant pH: sequence pH 6 → pH 7 → addition of NaCl, KCl, NH_4_Cl, CaCl_2_ (physiological levels) → pH 8.

The sensor integrates a PANi working electrode and an Ag/AgCl reference on permeable substrates, enabling saliva-through, potentiometric pH sensing with minimal dead volume (Figure 2A). Mechanistically, pH modulates the protonation state of PANi (emeraldine base ⇄ salt), shifting its interfacial potential relative to Ag/AgCl with an expected near-Nernstian slope. The top-view layout, with a circular sensing pad and a separated reference connected by low-resistance traces, minimizes ohmic drops and shields the reference from local acidity gradients during flow or capillary imbibition. Calibration across pH 4–10 is markedly linear (Figure 2B). The potential decreases monotonically with increasing pH, yielding an average slope of 49 mV pH^−1^ (group-to-group range ∼48–50 mV pH^−1^). Inter-measurement scatter (error bars) is intentionally displayed to reflect realistic intra-oral variability (saliva composition, temperature, and microflow); nevertheless, a linear fit across all replicates gives R^2^ ≈ 0.996–0.999, indicating that chemical drift and junction artifacts are negligible. The intercept near pH 7 is device-specific (200 ± 10 mV in our sets) and is governed by the PANi redox state after conditioning; it remains stable after several wet/dry and pH excursions (vide infra). Next, to address reproducibility, we fabricated five independent batches of sensors under the same processing conditions. Statistical analysis of the potential at each pH point (*n* = 5 batches) shows small variability (Figure S1). Dynamic staircase experiments establish reversibility and small hysteresis (Figure 2C). When pH is stepped every 3 min from 4→10 then 10→4 (total 60 min), the potential follows each step within seconds and overlays on the return sweep with a hysteresis area below the plot’s symbol size. Fitting the transients to a first-order response yields a characteristic time τ ≈ 8–10 s, consistent with rapid proton exchange and short diffusion paths through the porous stack. A complementary cycling stability protocol between pH 5 and pH 9 (6-min period; ten cycles in 60 min) shows high-fidelity square-wave tracking without amplitude loss (Figure 2D) Extended stability tests over 3 days, with repeated measurements every 6 h at pH 5 and pH 9, reveal only minor potential drifts (<10 mV) across 72 h, indicating that the permeable TPU/AgNW/PANi architecture maintains a stable potentiometric response under repeated use and saliva-like conditions, consistent with realistic intraoral usage patterns (Figure S2). The peak-to-peak potential remains constant over the ten cycles (retention >98%), and baseline wander is <1 mV despite repeated polarity switching. Such endurance under rapid polarity alternation—protonation at low pH and deprotonation at high pH, suggests that the volumetric swelling of PANi is modest and elastically recoverable within our thickness range; likewise, the Ag/AgCl reference maintains a stable chloride activity under the tested ionic strengths. Long-term stability (Figure 2E) was evaluated at fixed pH values of 4, 6, 8, and 10 over 12 h with 5-min sampling. All traces exhibit a short initial settling (∼30 min) followed by extremely slow linear drift of ≤ ±0.6 mV over 12 h. The drift is comparable to the thermal/electronic noise floor of benchtop potentiometric readouts and two orders of magnitude smaller than a one-pH-unit change (≈49 mV), providing a signal-to-drift ratio >80 for multi-hour monitoring. The absence of drift up-regulation at the extremes (pH 4 and 10) further indicates that neither over-oxidation (acid) nor over-deprotonation (alkali) processes accumulate during these holds, aligning with the reversible behavior seen in the staircase and cycling tests. Moreover, selectivity against dominant salivary electrolytes was examined by holding at pH 6 → pH 7, then sequentially spiking NaCl, KCl, NH_4_Cl, and CaCl_2_, before stepping to pH 8 (Figure 2F). As expected for a proton-responsive PANi/AgCl pair, ionic strength perturbations at constant pH induced only sub-millivolt to few-millivolt offsets (Na^+^ ≈ +0.5 mV; K^+^ ≈ +1.0 mV; Ca^2+^ ≈ +1.0 mV), with NH_4_^+^ giving the largest but still small excursion (≈+2.0 mV), likely reflecting weak acid-base interactions of ammonium at the film/solution interface. Intraoral temperature is typically tightly regulated around 36°C–37°C, with only modest short-term fluctuations. Given the measured temperature coefficient of ∼1–2 mV °C^−1^ at pH 7, such small variations are expected to induce only minor potential shifts (<5 mV), which are significantly smaller than the ≈50 mV change produced by a 1 pH unit step (Figure S3). More importantly, at pH 7, bending the device from a nearly flat state (radius 50 mm) down to 1 mm resulted in negligible changes in potential (<3 mV), indicating that curvature-induced mechanical stress has little effect on the electrochemical response. Under repeated bending at a radius of 5 mm for up to 5000 cycles, the potential at pH 7 showed only a minor drift of ∼4 mV, confirming excellent mechanical robustness and reliability under cyclic deformation (Figure S4). Collectively, the data show that the device delivers (i) near-Nernstian sensitivity across a clinically relevant range, (ii) fast, symmetric kinetics with negligible hysteresis under minute-scale pH fluctuations, (iii) excellent multi-hour stability with sub-millivolt drift, and (iv) high selectivity to pH over abundant salivary cations.

### Antibacterial performance of different materials

**Figure 3:**
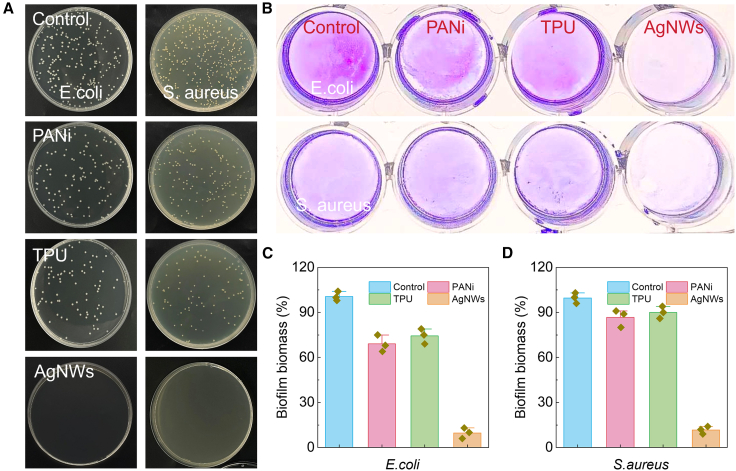
Antibacterial performance of different materials (A) Representative agar plate photographs of *E. coli* (left) and *S. aureus* (right) cultured on control, PANi, TPU, and AgNW. (B) Crystal violet staining assays of biofilm formation for *E. coli* (top) and *S. aureus* (bottom) confirm similar trends. (C and D) Quantitative analysis of bacterial viability relative to control for E. coli (blue) and S. aureus (red).

The antibacterial performance of the different permeable materials was systematically investigated against both Gram-negative Escherichia coli (E. coli) and Gram-positive Staphylococcus aureus (S. aureus). The control groups displayed extensive bacterial colonies, confirming robust bacterial proliferation in the absence of antimicrobial intervention (Figure 3A). Both PANI and TPU exhibited negligible bactericidal activity, with colony counts and biofilm staining comparable to the controls, underscoring their limited intrinsic antibacterial capacity. Evidentially, the AgNW network demonstrated a pronounced suppression of bacterial growth. The agar plate assays revealed almost complete inhibition of colony formation for both E. coli and S. aureus, while crystal violet staining further corroborated the suppression of biofilm formation, with only minimal residual adherence observed. Quantitative analysis highlighted the superior bactericidal efficacy of AgNWs, with bacterial viability reduced to near-background levels, whereas PANI and TPU only modestly mitigated proliferation (Figure 3B). The observed antimicrobial effect of AgNWs can be attributed to their mechanism of the release of silver ions, which interfere with cellular metabolism and induce oxidative stress. Thus, the integration of AgNWs into the sensing platform affords not only low-impedance electrical readout but also introduces intrinsic antimicrobial functionality. This dual role is critical for long-term stability and biofouling resistance in bioelectronic devices operating in complex physiological environments. By contrast, PANI and TPU, while mechanically robust and biocompatible, lack inherent antimicrobial features and thus require functional complementation. Quantitative analysis of bacterial viability relative to control for *E. coli* (blue) and *S. aureus* (red), highlighting the broad-spectrum antimicrobial efficacy of AgNWs (Figures 3C and 3D). Silver nanowires have been widely reported to maintain long-term antibacterial activity due to their sustained Ag^+^ release kinetics and structural stability under repeated hydration/dehydration cycles. Several studies have shown that AgNW-based films retain antibacterial performance after prolonged immersion or after multiple wet/dry cycles, supporting their applicability in dynamic oral environments. These literature reports, together with our own bactericidal and antibiofilm results, indicate that AgNWs embedded within the permeable TPU scaffold are capable of providing durable antimicrobial protection. Detailed comparison of our permeable and antimicrobial sensor with the existing pH sensors is listed in Table S1. Thus, these results demonstrate that permeable AgNWs network not only provides electrical functionality but also imparts intrinsic antibacterial activity, enabling stable and hygienic operation of oral application.

### Behavior-evoked oral pH dynamics *in situ*

**Figure 4:**
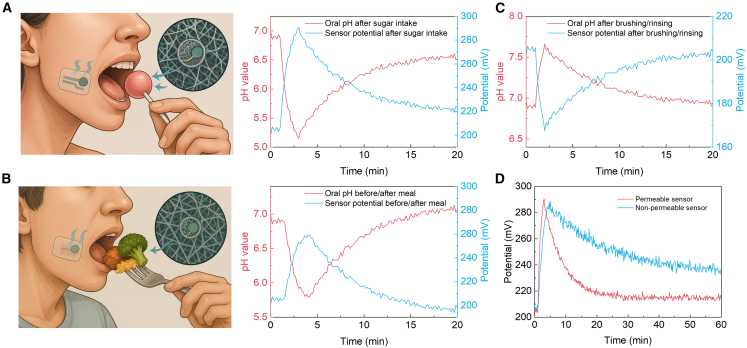
Behavior-evoked oral pH dynamics measured with a saliva-permeable potentiometric sensor (A and B) Illustrations of the in-mouth strip during sugar intake and a mixed meal, insets depict the breathable microstructure. Twenty-minute time courses of pH (left axis) and sensor potential vs. Ag/AgCl (right axis; red/blue traces as indicated). (C) Tooth-brushing/rinsing elicits a rapid alkaline excursion and a decay toward baseline. (D) Across scenarios, the permeable sensor preserves fast kinetics, enabling quantitative readout of acid burden and recovery.

Next, we fabricated saliva-permeable potentiometric sensors that couple fibrous TPU with a PANi working electrode and a co-planar Ag/AgCl reference. The breathable geometry ensures rapid saliva access while minimizing boundary-layer accumulation, enabling faithful capture of fast, behavior-evoked pH excursions. All traces are reported as pH (left axis) and the concomitant potential vs. Ag/AgCl (right axis). First, immediately after sucrose intake, the oral pH exhibits a canonical Stephan response, a rapid fall from ∼6.9 to a nadir near 5.0 within ∼3 min (ΔpH ≈ −1.9). A single-exponential fit gives 0.8 min, indicating acidification faster than typical salivary buffering. The potential increases concomitantly by ∼+90 mV, tracking the pH decrease without measurable lag. Recovery follows a slower exponential (5–6 min) and remains slightly acidic at 20 min (pH ≈ 6.6), consistent with ongoing plaque metabolism and incomplete acid clearance (Figure 4A). Second, a balanced meal produces a gentler but prolonged excursion: pH declines from ∼6.9 to ∼5.6, then rebounds past neutrality under stimulated salivary flow, tending to ∼7.1–7.2 by 20 min. The potential rises by ∼+64 mV to the nadir and subsequently drops below the baseline by ∼5–10 mV, mirroring the mild alkaline overshoot (Figure 4B). Third, exposure to an alkaline rinse yields the opposite polarity: pH rises from ∼6.9 to ∼7.8 and returns to the pre-event level with negligible drift, implying that surfactants/remineralizers do not foul the sensing interface over the tested window (Figure 4C). More importantly, head-to-head measurements after a standardized sugar intake revealed strikingly different trajectories for saliva-permeable versus non-permeable sensors. The permeable architecture (saliva-through TPU/AgNWs/PANi) recorded a fall from pH 6.9 to 5.05 within 3.0 min, then recovered to 6.7 at 60 min; the concomitant potential rose by ∼+90 mV at the nadir and relaxed toward baseline without residual drift. By contrast, the non-permeable sensor showed a deeper, slower excursion, pH 6.9 to 4.9 in 4.5 min, followed by a sluggish recovery that reached only 6.3 at 60 min, with the potential remaining elevated (∼+20–25 mV) long after the challenge (Figure 4D). These disparities are readily rationalized by mass transport. The permeable stack enables rapid convective-capillary access of bulk saliva to the sensing interface, shortening both the nadir residence and the recovery constant. The non-permeable encapsulation, in contrast, traps a stagnant film and supports local pH gradients, yielding deeper minima, pronounced hysteresis, and persistent offsets. Thus, these results establish saliva-permeable electronics as essential for accurate, low-drift, real-time oral pH sensing *in vivo*, enabling robust quantification of acid burden and recovery kinetics for caries-risk stratification and behavioral feedback.

In this work, we reported a saliva-permeable, antibacterial potentiometric strip that enables continuous, in-mouth pH monitoring with laboratory-grade fidelity. The porous TPU scaffold, percolated AgNW conductor, and PANi working electrode paired with an on-board Ag/AgCl reference preserve high mass transport (liquid/air permeability) while providing near-Nernstian sensitivity (∼49 mV pH^−1^), sub-minute kinetics, and minimal hysteresis. Long-term holds (12 h) show sub-mV drift, and salt additions at constant pH yield only millivolt-scale, reversible offsets, confirming functional proton selectivity. The AgNW layer contributes antibacterial protection without sacrificing transport or signal stability. Across calibrated challenges, the device resolves behavior-specific dynamics: a deep, rapidly developing Stephan drop after sugar, a moderate meal-induced excursion with alkaline rebound, and a fast alkaline rise after brushing/rinsing. This work establishes a practical route to continuous oral pH sensing suitable for caries-risk stratification, hygiene feedback, and materials/therapeutics evaluation.

### Limitations of the study

Although the saliva-permeable potentiometric strip shows stable calibration, low drift, and feasibility in short intraoral recordings, several limitations remain. Human-use tests primarily validated time resolution during common daily events; broader clinical translation will require larger, more diverse cohorts and longer wear durations across different oral-health states, diets, and salivary-flow conditions. Measurements represent the local saliva environment at the sensor site rather than plaque pH at the tooth-biofilm interface, and spatial gradients or placement may affect absolute values and kinetics. Because the device uses an on-board Ag/AgCl reference and a PANi working electrode, changes in temperature, chloride activity, and ionic strength may introduce offsets, motivating standardized calibration and future temperature compensation. While antimicrobial assays show the inhibition of selected organisms, long-term performance under multispecies biofilms, protein adsorption, food residues, and mechanical abrasion remains to be established. Finally, Ag-nanowire-mediated antibacterial activity warrants extended biocompatibility and silver-release assessments, along with integration toward untethered readout.

## Resource availability

### Lead contact

Requests for further information and resources should be directed to and will be fulfilled by the lead contact, Feng Yang (yangfeng@xzhmu.edu.cn).

### Materials availability

This study did not generate new unique reagents.

### Data and code availability

The data supporting the findings of this study are available within the article and the supplemental information. This study does not generate original code. Any additional information required is available upon reasonable request to the lead contact.

## Acknowledgments

This article was supported by the Special Fund for Staff Overseas (Cross-Border) Research and Training of the Affiliated Hospital of Xuzhou Medical University and Proof-of-Concept Project of Xuzhou Medical University (number: GNYZ2024005).

## Author contributions

Conceptualization, L. Z. and F.Y.; methodology, L.Z. and M.W.; investigation, L.X. and Z.S.; writing-original draft, L.Z.; writing-review and editing, F.Y.; funding acquisition, F.Y.; resources, X.L.; supervision, F.Y.

## Declaration of interests

The authors declare no competing interests.

## STAR★Methods

### Key resources table

**Table:** 

REAGENT or RESOURCE	SOURCE	IDENTIFIER
Thermoplastic polyurethane	Foster Corporation	CAS: 9009-54-5
Silver nanowires	XFNANO	CAS: 7440-22-4
Polyaniline	Aladdin	CAS: 25233-30-1
Dimethylformamide	Sigma Aldrich	CAS: 68-12-2
N-methyl-2-pyrrolidone	Sigma Aldrich	CAS: 872-50-4
Tetrahydrofuran	Sigma Aldrich	CAS: 109-99-9
Ag/AgCl paste	Sigma Aldrich	CAS: 7783-90-6

### Experimental model and study participant details

Human participant procedures were approved by the Ethics Committee of Xuzhou Medical University (approval no. XYTY2024 KL210-02). All procedures conformed to institutional guidelines. Written informed consent was obtained from all participants prior to enrollment. Participants were informed about the study purpose, procedures (including intraoral placement of the pH sensor strip), potential risks (e.g., temporary discomfort or irritation), and their right to withdraw at any time without penalty. Two healthy volunteers were enrolled (both male; ages 26 and 28 years).

### Method details

#### Fabrication of pH sensor

Electrospun TPU fibers were prepared as follows. TPU (1.5 g) was dissolved in 10 mL of DMF/THF (v/v = 1:1) and stirred 8 h to obtain a homogeneous spinning dope. The solution was loaded into a syringe equipped with an 18 G metal needle and electrospun at 4 mL h^-1^ for 2.5 h with a tip-to-collector distance of 10 cm and an applied voltage of 20 kV. Fibers were collected on an Al-foil-wrapped rotating drum (200 rpm). Conductive/antibacterial networks were formed by spray-coating AgNWs onto the TPU fiber. The overall TPU substrate size was 12 mm × 12 mm, and the patterned AgNW electrode had a width of 3 mm. The pH-sensitive working electrode was defined by drop-casting a PANi solution onto the designated circular sensing area and drying to form a conformal film (diameter 4 mm). The on-board Ag/AgCl reference electrode with a diameter of 4 mm was patterned with Ag/AgCl paste and thermally dried.

#### Material and sensor characterization

Surface morphology were imaged by SEM (Zeiss Compact, 3 kV), elemental mapping used EDS under 15 kV. Electrospun TPU, AgNWs and PANi films were conditioned at 20 °C (50% RH) for ≥12 h, then tested for air permeability and water evaporation in 12-h series with 30-min sampling. Air permeability followed ASTM D737 on a constant ΔP rig (ΔP = 125 Pa) using a circular test head; the steady volumetric flow at the orifice was averaged and normalized by the effective area to report J_air_ in L·m^-2^·s^-1^. Water evaporation used an upright cup per ASTM E96 with deionized water or artificial saliva sealing the cup beneath the specimen; cups were held at 20 °C (50% RH), weighed every 30 min, and the steady mass-loss rate divided by area reported as mg·cm^-2^·h^-1^. Electrochemical measurements (open-circuit potential vs Ag/AgCl, calibration, stability) were performed using an electrochemical workstation (CHI760E) with temperature-controlled regions when specified. Before each set of measurements, the permeable PANi-based pH sensor was calibrated in a series of standard aqueous buffer solutions spanning the physiologically relevant oral pH range, all calibration measurements were performed at room temperature.

#### Bacterial biofilm biomass detection

The antimicrobial activity of E. coli and S. aureus were assessed through biofilm biomass quantification using the crystal violet staining method. Briefly, bacterial suspensions were seeded into 24-well plates and incubated at 37 °C for 24 h under static conditions to promote biofilm formation. Following incubation, non-adherent cells were removed by gentle PBS rinsing, while adherent biofilms were fixed and subsequently stained with 0.1% crystal violet for 15 min at room temperature. Excess dye was removed by thorough PBS washing, and the bound stain was eluted using 95% ethanol. The absorbance of the solubilized crystal violet was recorded at 595 nm, and biofilm biomass was quantified using ImageJ analysis.

### Quantification and statistical analysis

The statistical details of the experiments are presented in Figures 1, 2, 3, and 4 were analyzed using Microsoft Excel, and Origin. The corresponding n value for Figure 2 is six, meaning that each pH response result was derived from six samples. The terms center and dispersion refer to the mean and standard deviation, respectively.

#### Additional resources

Clinical trial registration: Not applicable. No clinical trial registry number is available because this study did not involve a registered clinical trial.
