# Supplementary material for: Saliva-permeable and antimicrobial potentiometric pH sensor for oral health monitoring
Source: iScience. 2026 Jan 15;29(2):114703. doi: 10.1016/j.isci.2026.114703 (PMC12887250; doi:10.1016/j.isci.2026.114703)
Supplement: Document S1. Figures S1–S4 and Table S1 [file mmc1.pdf]

iScience, Volume *29*

Supplemental information

Saliva-permeable and antimicrobial potentiometric pH sensor for oral health monitoring

Luyue Zhang, Mengya Wang, Liting Xiao, Xinchang Li, Ziqi Sha, and Feng Yang

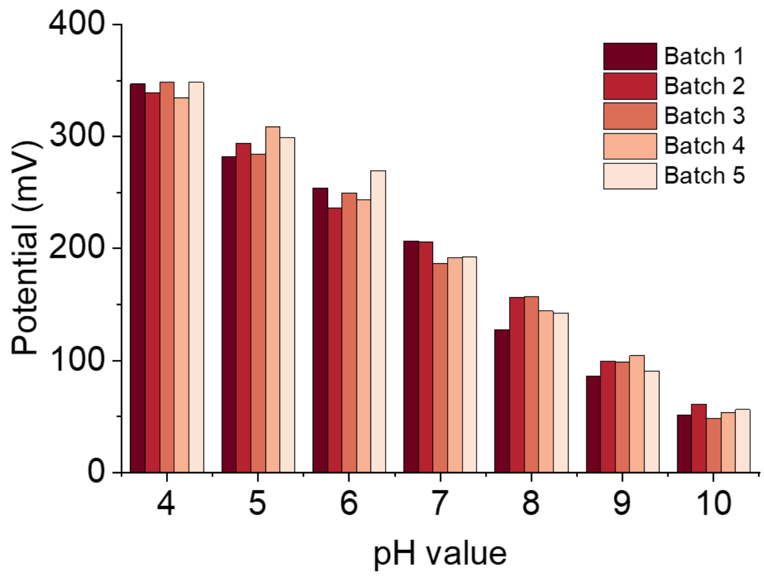

Figure S1: Batch-to-batch reproducibility of the permeable pH sensor. Potentials measured at pH 4-10 for five independently fabricated sensor batches (Batch 1 to Batch 5). All batches exhibit consistent potentiometric responses with minimal variation across the full pH range, demonstrating excellent fabrication reproducibility of the TPU/AgNW/PANi permeable architecture.

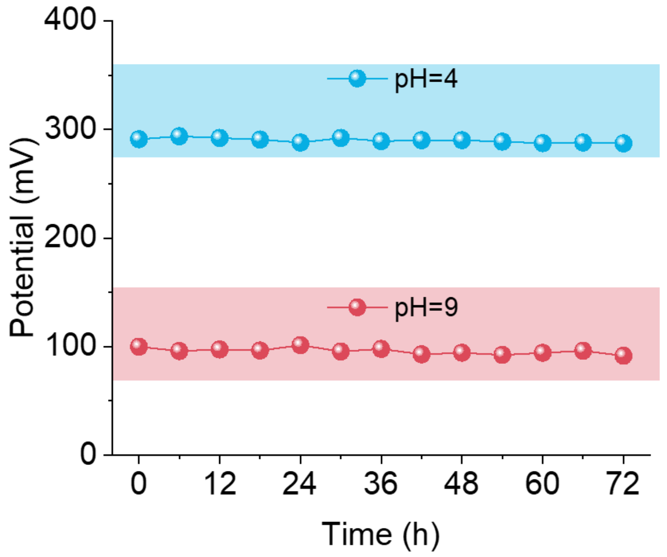

Figure S2: Long-term stability of the permeable pH sensor over 72 hours. Time-dependent potentials recorded at pH 4 (blue) and pH 9 (red) under continuous measurement. The shaded regions represent the full potential fluctuation range for each pH condition. Over 72 h, the sensor exhibits only minimal drift, demonstrating excellent long-term stability and suitability for prolonged intraoral monitoring.

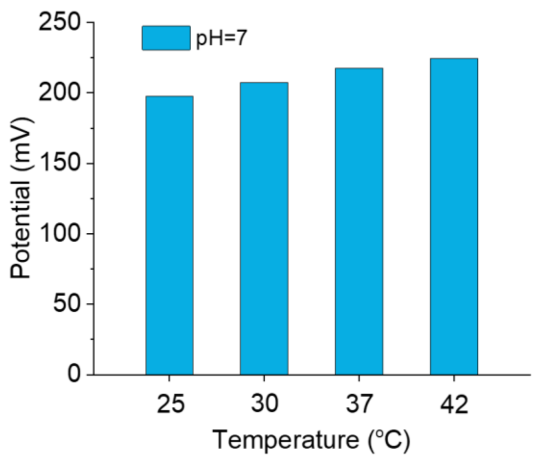

Figure S3: Temperature dependence of the permeable pH sensor at pH 7. Potential measured at four controlled temperatures (25, 30, 37, and 42 °C). The potential exhibits a modest, monotonic increase with temperature, corresponding to a temperature coefficient of approximately 1-2 mV $°C^{-1}$. This small variation indicates that normal intraoral temperature fluctuations have minimal impact on sensing accuracy.

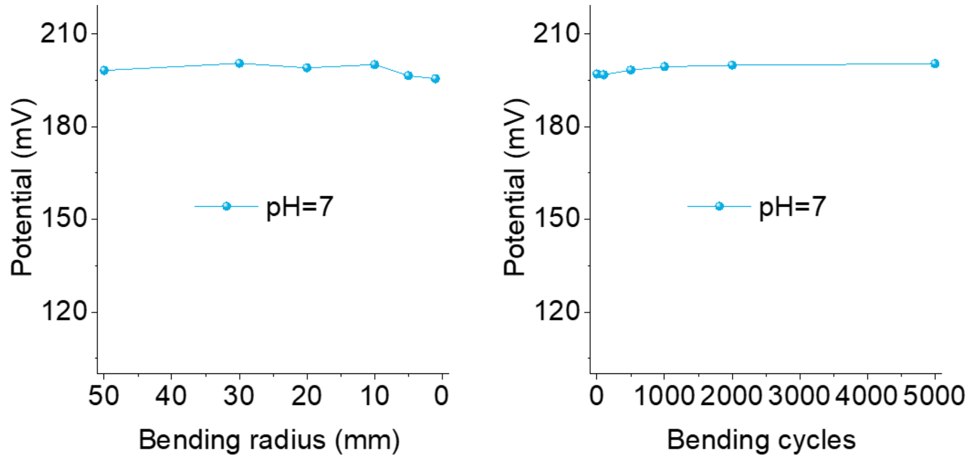

Figure S4: Mechanical robustness of the permeable pH sensor under bending deformation. (left) Potential measured at pH 7 while bending the sensor over radii ranging from 50 mm to 1 mm. The potential remains highly stable (<3 mV variation), indicating negligible influence of curvature-induced strain. (right) Potential response at pH 7 during repeated bending at a fixed radius of 5 mm for up to 5000 cycles. Only minimal drift (~4 mV) is observed, demonstrating excellent mechanical durability and reliability of the TPU/AgNW/PANi permeable architecture under cyclic deformation.

Table S1: Detailed comparison of our permeable and antimicrobial sensor with the existing pH sensors.

Materials	Sensitivity	Durability	Selectivity	Response	Properties	Ref
PANI /gold fiber	60.6 mV/pH	8000 s	NH_4^+ Ca^{2+} Mg^{2+} Na^+	/	Stretchable	1
PANI-rGO	38 mV/pH	/	NH_4^+ K^+ Na^+	50 s	Wearable	2
PANI/$Ti_3C_2T_x$	43.52 mV/pH	1800 s	NH_4^+ Ca^{2+} Mg^{2+} Na^+ K^+	/	Wearable	3
PANI/SPCE	67.53 mV/pH	/	Ca^{2+} Mg^{2+} Na^+ K^+	2 s	Skin-Attachable	4
PANI/CNT	55.87 mV/pH	/	NH_4^+ Ca^{2+} Mg^{2+} Na^+ K^+	/	Wearable	5
PANI	48.8 mV/pH	72 hours	NH_4^+ Ca^{2+} Na^+ K^+	8 s	Permeable, antimicrobial	This work

References:

[1] Wang, Ren, et al. "Stretchable gold fiber-based wearable electrochemical sensor toward pH monitoring." Journal of Materials Chemistry B 8.16 (2020): 3655-3660.
[2] Mazzara, Francesca, et al. "PANI-based wearable electrochemical sensor for pH sweat monitoring." Chemosensors 9.7 (2021): 169.
[3] Chen, Lijuan, et al. "Superhydrophobic functionalized $Ti_3C_2T_X$ MXene-based skin-attachable and wearable electrochemical pH sensor for real-time sweat detection." Analytical Chemistry 94.20 (2022): 7319-7328.
[4] Zhu, Chonghui, et al. "A dual-functional polyaniline film-based flexible electrochemical sensor for the detection of pH and lactate in sweat of the human body." Talanta 242 (2022): 123289.
[5] Wang, Lie, et al. "A core–sheath sensing yarn-based electrochemical fabric system for powerful sweat capture and stable sensing." Advanced Functional Materials 32.23 (2022): 2200922.
